# Effect and process evaluation of a preschool-based intervention to promote an early childhood education and care teacher-parent partnership about healthy behaviours in children: Study protocol for the cluster randomised controlled trial CO-HEALTHY

**DOI:** 10.1371/journal.pone.0281999

**Published:** 2023-02-22

**Authors:** Nicole Toussaint, Martinette T. Streppel, Sandra Mul, Meryem Gündüz, Marloes D. A. van Verseveld, Mirka Janssen, Peter J. M. Weijs, Ruben G. Fukkink

**Affiliations:** 1 Faculty of Sports and Nutrition, Center of Expertise Urban Vitality, Amsterdam University of Applied Sciences, Amsterdam, The Netherlands; 2 Department of Education and Lifelong Learning, Norwegian University of Science and Technology, Trondheim, Norway; 3 Department of Nutrition & Dietetics, Amsterdam University Medical Centers, VU University, Amsterdam, The Netherlands; 4 Faculty of Child Development and Education, Amsterdam University of Applied Sciences, Amsterdam, The Netherlands; 5 Faculty of Social and Behavioural Sciences, University of Amsterdam, Amsterdam, The Netherlands; St John’s University, UNITED STATES

## Abstract

**Background:**

Early Childhood Education and Care (ECEC) teachers at urban preschools are potential key figures to promote healthy behaviours in disadvantaged young children and to engage parents in lifestyle-related topics. An ECEC teacher-parent partnership regarding healthy behaviours may support parents and stimulate their children’s development. However, it is not an easy task to establish such a collaboration and ECEC teachers need tools to communicate with parents about lifestyle-related topics. This paper describes the study protocol of a preschool-based intervention (CO-HEALTHY) to promote an ECEC teacher-parent partnership regarding healthy eating, physical (in)activity and sleeping behaviours in young children.

**Methods:**

A cluster randomised controlled trial will be performed at preschools in Amsterdam, the Netherlands. Preschools will be randomly allocated to an intervention or control group. The intervention consists of a toolkit with 10 parent-child activities and associated training for ECEC teachers. The activities were composed using the Intervention Mapping protocol. At intervention preschools, ECEC teachers will carry out the activities during standard contact moments. Parents will receive associated intervention materials and will be encouraged to perform similar parent-child activities at home. At control preschools, the toolkit and training will not be implemented. The primary outcome will be the teacher- and parent-reported partnership regarding healthy eating, physical (in)activity and sleeping behaviours in young children. The perceived partnership will be assessed by a questionnaire at baseline and at 6 months. In addition, short interviews with ECEC teachers will be held. Secondary outcomes include the knowledge, attitude, food- and activity-related practices of ECEC teachers and parents. Furthermore, children’s eating, physical (in)activity and sleeping behaviours, and weight development will be assessed. A process evaluation of the intervention will be made.

**Discussion:**

The intervention aims to provide a practical tool for ECEC teachers at urban preschools to promote an ECEC teacher-parent partnership regarding a healthy lifestyle in young children.

**Trial registration:**

Netherlands Trial Register (NTR): NL8883. Date registered: September 8, 2020.

## Introduction

Healthy eating, physical (in)activity and sleeping behaviours are important lifestyle factors for a normal weight development in children [1]. Particular among children from families with diverse ethnic backgrounds and/or low socioeconomic positions, excess weight gain and unhealthy behaviours in children are still common [2,3]. Children show health inequalities already at a young age and the need for early interventions to promote healthy behaviours is widely recognised [4–8].

In the Netherlands, urban preschools are environments in which young children (2 to 4 years old) with disadvantaged backgrounds can be reached. The preschools provide play-based education and prepare children for primary school. Children generally spend up to 16 hours per week in preschool and this setting is therefore an important environment for early interventions in deprived areas to overcome health discrepancies [9,10].

Early Childhood Education and Care (ECEC) teachers at urban preschools are potential key figures to promote healthy behaviours and engage parents in lifestyle-related topics [11,12]. In the context of promoting a healthy lifestyle in young children, a partnership between ECEC teachers and parents is desired. Parental engagement makes a transfer from the ECEC setting to the home environment possible. Ward et al. (2017) describe in their systematic review on strength of obesity prevention interventions in ECEC settings that parental engagement improves the effect of interventions. The studies included in their review were mainly conducted in ECEC settings with many participants who had low to middle socioeconomic positions [13]. Furthermore, van der Kolk et al. (2019) report in a systematic review that interventions in ECEC settings with parental involvement show promising outcomes on health-related behaviours. However, the authors emphasise that evidence is limited, in particular for anthropometric outcomes. They also discuss the issue of possible selection bias in interventions using parental involvement, as parents’ socioeconomic position is associated with participation in these interventions [14]. Flynn at al. (2022) describe in a recent systematic review that interventions in the home setting and interventions which included parents were effective in preventing childhood obesity [15].

Engaging parents to stimulate healthy behaviours in children is not an easy task. Dev et al. (2017) reported, based on their qualitative study on perspectives of ECEC teachers in communicating with parents about nutrition and health, various barriers to engage parents: parents are too busy, ECEC teachers are unsure of how to communicate about nutrition without offending parents, and teachers are concerned whether parents use nutrition educational materials. Reported successful strategies for communication included building a partnership with parents through parent education and recognising the benefits of communicating with parents about nutrition to support children’s health [16]. Jayasuriya et al. (2016) performed a descriptive exploratory study on parents’ perception of physical activity at preschool. They suggest the need for more effective communication between ECEC teachers and parents, as parents have little knowledge about outdoor playtime, outdoor clothing and weather policies of ECEC centres [17]. Furthermore, Oakes et al. (2020) emphasise that there are significant limitations in frequency and content of ECEC teacher-parent communication about children’s sleep. Frequent and two-way communication between ECEC setting and families can help to build partnerships that support children’s sleeping needs [18]. However, ECEC teachers indicate that they need tools to communicate with parents about healthy lifestyle-related topics [19].

In this study, the effects of a preschool-based intervention (CO-HEALTHY) for ECEC teachers will be examined. The intervention consists of a toolkit and associated training for ECEC teachers, complemented with related intervention materials for parents at home. The materials are simple and culturally sensitive images are used to reach families with different sociocultural and socioeconomic backgrounds. This study primarily aims to examine the effects of the preschool-based intervention on the ECEC teacher-parent partnership regarding eating, physical (in)activity and sleeping behaviours in young children (2 to 4 years old). As the parent-child activities also intend to achieve a transfer to the home environment (behavioural change in parents), our second aim is to examine the effects of the intervention on parents’ knowledge, attitude and food- and activity-related practices, children’s eating, physical (in)activity and sleeping behaviours, and children’s weight development.

## Materials and methods

### Study design and setting

The present study concerns a cluster randomised controlled trial. Urban preschools of 3 large childcare organisations in Amsterdam, the Netherlands, will be randomly allocated to an intervention or control group. In total, 60 preschools are available for randomisation. The preschools are mainly located in the city districts Zuidoost, Nieuw-West and Noord. These areas are characterised by a relatively high level of families with diverse ethnic backgrounds and/or low socioeconomic positions. In 2018, the prevalence of overweight and obesity among 3-year-olds was respectively high at 12.1%, 11.3% and 9.6% for the districts Zuidoost, Nieuw-West and Noord, whereas in other districts of Amsterdam this percentage ranged from 5.6% to 8.7% [20].

**Fig 1:**
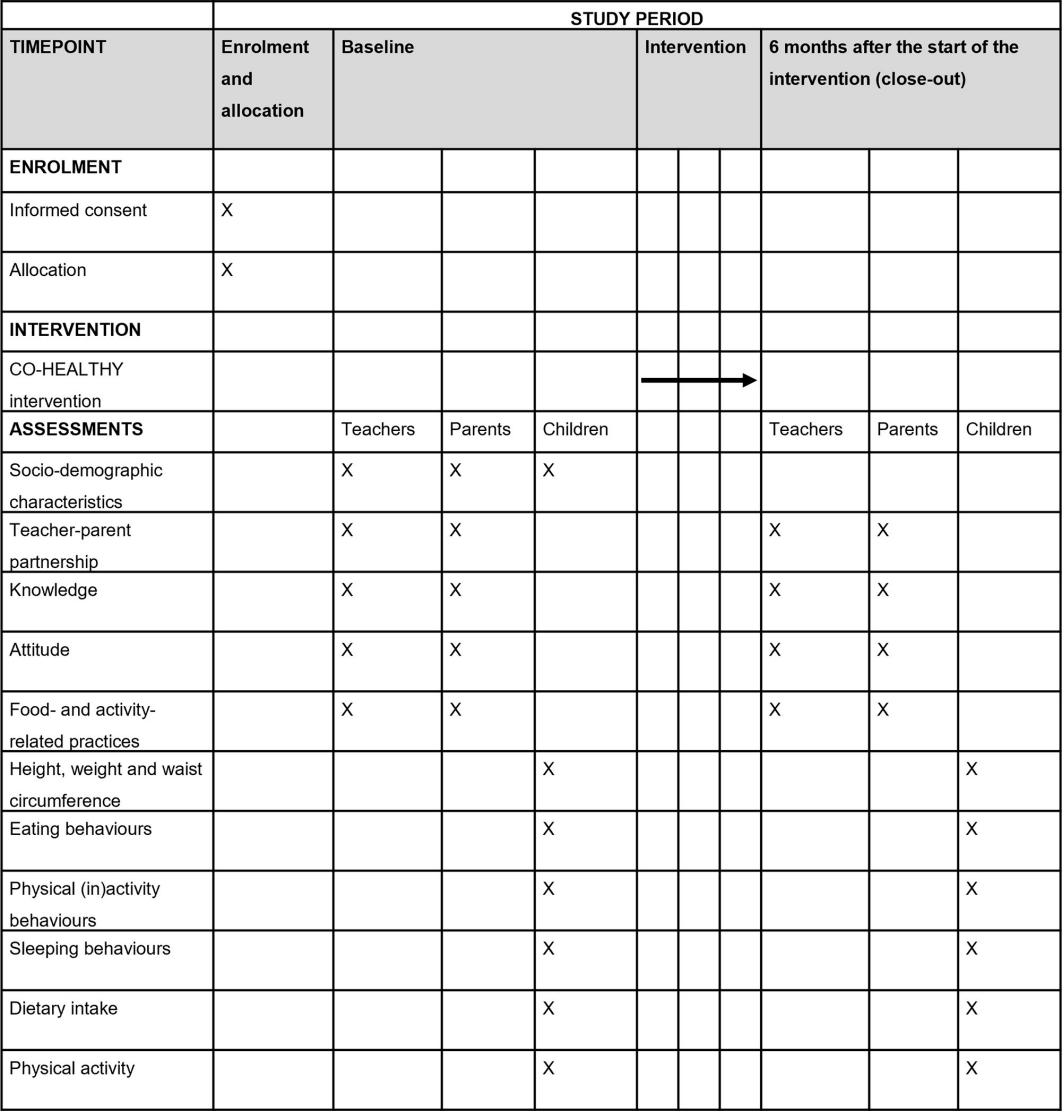
SPIRIT statement with schematic overview of the study.

The study period for each preschool is 6 months. Data of ECEC teachers, parents and children will be collected at baseline and at 6 months. After the baseline measurements, the CO-HEALTHY intervention will be carried out at preschools in the intervention group. The intervention consists of a toolkit and associated training for ECEC teachers to promote the ECEC teacher-parent partnership regarding healthy eating, physical (in)activity and sleeping behaviours in young children. The toolkit includes 10 parent-child activities. At control preschools, the toolkit and associated training for ECEC teachers will not be implemented. However, we will provide 5 toolkits to each childcare organisation upon completion of the study so that they can distribute it among locations in the control group. Fig 1 shows a schematic overview of the study. Protocol version 1 was registered in the Netherlands Trial Register on September 8, 2020, prior to the start of the study. The Institutional Review Board of VU University Medical Center in Amsterdam reviewed and approved the study on August 25, 2020 (2020.279 –NL73907.29.20). Recruitment began on September 28, 2020 and the study is ongoing (with interruptions and challenges due to COVID-19).

### Intervention design

The intervention consists of a toolkit and associated training for ECEC teachers. At intervention preschools, at least 1 ECEC teacher will follow a 3-hour training session delivered by 2 researchers of the Amsterdam University of Applied Sciences (AUAS) in small training groups. The training focusses on the teachers’ knowledge and skills to collaborate with parents regarding lifestyle behaviours of their children. The first part of the training presents theory about collaborating with parents. ECEC teachers will be invited to share their own experience in collaborating with parents about lifestyle related topics: what works and what does not work in daily practice. Also, a case from daily practice will be discussed and some practical tips will be shared. In the second part of the training, the toolkit with 10 parent-child activities will be presented and theory about healthy eating, physical (in)activity and sleeping behaviours in young children will be discussed. Together, the toolkit and training provide a practical guide for ECEC teachers at urban preschools to communicate with parents about lifestyle-related topics. A reader with information from the training will be provided to the teachers. After the training session, the teachers will communicate the content of the toolkit and training to their local team of colleague teachers who were not able to follow the training session. The parent-child activities, of about 15 minutes each, will be carried out by ECEC teachers during standard contact moments at preschool (at the walk-in play time/when parents bring their child). This procedure is practical for parents as they do not have to invest additional time for the activities. ECEC teachers will be asked to perform at least the activities related to healthy eating, playing physical activity games together and sleeping (to cover the lifestyle themes) plus to perform 3 other activities of which they think suit their parents and children the most. Parents will receive congruent intervention materials per activity and will be encouraged to perform parent-child activities at home. An example of a parent-child activity is a game with bedtime routine cards. In the toolkit there are cards with images of activities parents/children can do at home every night before the child is going to sleep (i.e. brushing teeth). Parents and children can cut out the bedtime routine cards and put them in an order to create a bedtime routine. The cards can be glued on a sheet and this sheet can be taken at home so that the cards can be used at home. Parents will receive an information card with some practical advice about sleeping (i.e. “do the same things with your child every night before going to sleep”). The intervention materials are simple and culturally sensitive images are used. Furthermore, the materials will be provided in the 4 most spoken languages in the city districts: Dutch, English, Turkish and phonetic Arabic. The proposed parent-child activities at home are familiar for parents and children as they practiced a similar activity at preschool. In the training and reader for ECEC teachers it will be highlighted to ask parents about the activities at home. Furthermore, the toolkit contains a poster and stickers for in the classroom (together, children and parents can collect as many stickers as possible by putting stickers at the poster when doing activities at home).

### Intervention mapping

The Intervention Mapping protocol is a systematic approach to plan health promotion programs [21,22], which we applied to develop the parent-child activities from the CO-HEALTHY intervention. The protocol involves 6 steps.

In the first step, a logic model of the problem was created by identifying factors that cause or influence excess weight gain in preschool children. A planning group with important stakeholders was established and included ECEC teachers, parents, representatives of renowned Dutch knowledge centres on different aspects of lifestyle, the national organisation for parents in ECEC, the national organisation for teachers in ECEC, dietitians and the Public Health Service of Amsterdam.

Next, a needs assessment using the PRECEDE model [23] was conducted. Interviews with experts (n = 9), a literature study, focus groups with ECEC teachers in the different city districts (n = 10 teachers) and planned conversations with parents (n = 2 parents) served as input for the PRECEDE model. The research staff experienced that only parents who were already involved in a healthy lifestyle for their child wanted to participate in a planned conversation. Therefore, the research staff switched to another strategy. They visited a preschool 3 times (at 2 different childcare organisations) and asked a multicultural group of parents (n = 28) to identify healthy lifestyle topics for their child which they think are easy or difficult to pursue. The parents could put a green (for the easy ones) or yellow sticker (for the difficult ones) at a predefined list of healthy lifestyle-related topics. As the children (2–4 years) are mainly dependent of their parents, we choose to focus in the PRECEDE model only on the behaviours of parents.

ECEC teachers indicated that the activities should be fun, accessible, practical in their execution and should not cost extra time. Based on the focus groups, expert interviews and conversations with parents, in total 10 important behaviours were distinguished: 1) parents do not provide a breakfast or provide an unhealthy breakfast, 2) parents do not involve their child in meal planning and preparation, 3) parents provide too many unhealthy foods and too little healthy foods, 4) parents provide too large portion sizes, 5) parents do not provide water, 6) parents do not play/are not physically active together with their child, 7) parents allow too much screen time, 8) parents give their child too little opportunities to be physically active, 9) parents do not let their child play outside, and 10) parents do not ensure their child get enough sleep. At the end of the needs assessment, programme objectives were described (Table 1).

**Table 1:** Overview of formulated programme and performance objectives for preschoolers’ parents.

Target Group	Programme Objectives	Performance Objectives (PO)
Parents	1. Parents increase the number of days per week the child eats (a healthy) breakfast	PO.1.a. Parents facilitate a healthy breakfast at home
		PO.1.b. Parents stimulate the child to eat a healthy breakfast
		PO.1.c. Parents make sure there is enough time for the child to eat a healthy breakfast
	2. Parents involve the child in meal planning and preparation more often	PO.2.a. Parents facilitate that the child can help in meal planning and preparation
		PO.2.b. Parents stimulate the child to help in meal planning and preparation
	2. Parents increase the number of days per week the child eats vegetables	PO.2.c. Parents stimulate the child’s vegetable intake
		PO.2.d. Parents are role models by eating healthy in front of the child
		PO.2.e. Parents talk to the child about the importance of healthy eating
	3. Parents increase the number of days per week the child eats healthy foods	PO.3.a. Parents provide fruit and vegetables every day
		PO.3.b. Parents provide water (instead of sugar-sweetened beverages)
		PO.3.c. Parents provide whole grain products instead of white products
		PO.3.d. Parents provide meals at set times (3 main meals and 1 or 2 healthy snacks)
		PO.3.e. Parents use soft and liquid fats instead of solid fats
		PO.3.f. Parents minimise the intake of candy, biscuits, snacks and soft drinks
		PO.3.g. Parents have healthy foods available at home
		PO.3.h. Parents do not reward, punish and comfort with foods
	4. Parents regularly provide appropriate portion sizes	PO.4.a. Parents adjust portion sizes to (age) appropriate sizes for the child
		PO.4.b. Parents do not tell the child to finish his/her plate
	5. Parents increase the number of days per week the child drinks water	PO.5.a. Parents stimulate the intake of water
		PO.5.b. Parents minimise the intake of soft drinks
	6. Parents play/are being physically active together with the child more often	PO.6.a. Parents facilitate that the child can play physical activity games with them
		PO.6.b. Parents participate in physical activity games
		PO.6.c. Parents are role models by being physically active themselves
	7. Parents let the child have screen time less often	PO.7.a. Parents stimulate the child to play inside without TV, computer, tablet or mobile phone
		PO.7.b. Parents set and follow rules about screen time
		PO.7.c. Parents do not allow screens during meal times
		PO.7.d. Parents do not allow screen time in the hour before going to bed
	8. Parents give the child more opportunities to be physically active	PO.8.a. Parents let the child be physically active when moving from place to place together with child
		PO.8.b. Parents stimulate the child to be physically active when moving from place to place
		PO.8.c. Parents let the child practice with a balance bike
	9. Parents increase the number of days per week the child plays outside	PO.9.a. Parents facilitate the child can play outside
		PO.9.b. Parents stimulate the child to be physically active outside in different ways
	10. Parents ensure a good sleep hygiene for the child more often	PO.10.a. Parents stimulate the child to get enough sleep
		PO.10.b. Parents provide/follow a bedtime ritual
		PO.10.c. Parents do not allow screen time in the hour before going to bed

In the second step, desired changes in the behaviour of parents were stated and the programme objectives were subdivided into performance objectives (the required actions for the health promoting behavioural outcomes) (Table 1). Next, personal determinants in parents were selected based on importance (strength of association with the behaviour) and changeability (how likely it is that the intervention is going to influence a change in the determinant). We concluded to focus on the determinants ‘knowledge’, ‘attitude’, ‘skills’ and ‘self-efficacy’. Thereafter, change objectives were formulated by crossing the performance objectives with the selected personal determinants. After formulating change objectives, we proceeded to step 3 (program design). In step 3, we selected theory-based methods for change in the personal determinants. Next, practical applications were designed to put the theory-based methods into practice (Table 2).

**Table 2:** Overview of the CO-HEALTHY applicants and their underlying theory-based methods to change the behavioural determinants of the preschoolers’ parents.

Determinants	Theory-based methods	Application in CO-HEALTHY intervention
Knowledge	Using imagery (Theories of Information Processing: Steen, 2007; Wright, 2012)	Images for eating healthy, playing physical activity games and getting enough sleep (e.g. image of happy child doing a physical activity together with parent on a physical activity calendar for parents/children).
	Advance organisers (Theories of Information Processing: Kools, 2012; Kool, van de Wiel, Ruiter, Crüts, & Kok, 2006)	Short and practical tips in reader for ECEC teachers and on information cards for parents (with each activity there is an information card for parents).
	Discussion (Elaboration Likelihood Model: Petty et al., 2009)	Training and reader for ECEC teachers emphasise to communicate with parents about a lifestyle theme during the parent-child activities at the walk in play time.
Attitude	Environmental re-evaluation (Trans-Theoretical Model: Prochaska et al., 2015)	Images of positive effects after healthy behaviours (e.g. image of full battery after having a healthy breakfast).
	Arguments (Communication-Persuasion Matrix; Elaboration Likelihood Model: McGuire, 2001; Petty & Wegener, 1998)	Arguments for benefits of healthy behaviour/cons of unhealthy behaviour on information cards for parents (e.g. tip on information card about playing outside “Let your child play outside often. Your child will be physically active in different ways. This is good for your child’s body.”).
	Cultural similarity (Communication-Persuasion Matrix: Kreuter & McClure, 2004)	Cultural specific animations and images of cultural specific healthy food products.
	Direct experience (Theories of Learning: Maibach & Cotton, 1995)	Guided parent-child activities during walk in play time. The proposed parent-child activities at home are recognisable for parents and children as they practiced a similar activity at preschool.
	Repeated exposure (Theories of Learning: Zajonc, 2001)	Repeated exposure to lifestyle themes in different parent-child activities and there are parent-child activities for the preschool and the home environment.
Skills & Self efficacy	Planning coping responses (Attribution Theory and Relapse Prevention Theory; Theories of Self-Regulation: Marlatt & Donovan, 2005)	Alternatives for unhealthy behaviours (e.g. tip on information card about healthy foods and drinks “Do not reward, punish and comfort with food. Rather give a hug or do something fun together.”).
	Guided practice (Social Cognitive Theory; Theories of Self-Regulation: Kelder et al., 2015)	With guided parent-child activities during walk in play time.
	Provide contingent rewards (Theories of Learning; Theories of Self-Regulation: Bandura, 1986)	Sticker cards for children (e.g. “Put a sticker on the back of this information card together with your child every time your child tastes a vegetable”.) and sticker poster in classroom (together, children and parents can collect as many stickers as possible by putting stickers at the poster when doing activities at home).
	Goal setting (Goal-Setting Theory; Theories of Self-Regulation: Latham & Locke, 2007)	Parents-child activities/assignments for the home environment.

Intervention materials were produced in close collaboration with the planning group in the fourth step. The materials were optimised after receiving feedback from the implementers (iterative process).

In step 5 we worked on an implementation plan. ECEC teachers will implement the intervention and a practical reader and training for ECEC teachers were designed.

Step 6 consisted of making a plan to evaluate the effects and process of the intervention. We planned to examine the effects of the intervention as described in this study protocol (via a cluster randomised controlled trial). Furthermore, we planned to interview ECEC teachers and parents in the intervention group as input for a process evaluation. It will concern semi-structured interviews for which an interview guide with open- and closed ended questions was made.

### Recruitment

The participating childcare organisations provided consent to involve all ECEC teachers, as further training for their employees, and carry out the parent-child activities at intervention preschools. Not all ECEC teachers and parents/children at intervention locations will also participate in the study as participation is voluntary.

All ECEC teachers at participating preschools will be informed about the study through their directors. Next, the AUAS research staff will contact ECEC teachers to give oral information about the study and hand out an information letter with Informed Consent form. One week later, the AUAS research staff will recontact the locations to answer any remaining questions and, if ECEC teachers wish to participate, obtain written Informed Consent. ECEC interns will be excluded from participation in this study.

Parents will get an announcement from ECEC teachers that AUAS researchers will soon visit their preschool. The research staff then will visit the locations at the walk-in play time when parents bring their child and will provide oral and written information. The parents will be asked to read the information letter and consider participation. The AUAS research staff will revisit the locations after one week and obtain written Informed Consent from parents who would like to participate in the study with their child. Parents will be asked to give written Informed Consent for themselves as well as for the participation of their child. All children are between 2 and 4 years old; in the Netherlands children generally go to primary school at the age of 4.

This extensive recruitment strategy seems necessary to reach the target population and include the required number of participants.

### Randomisation

The parallel group randomisation will be performed by a blinded researcher of the AUAS. It concerns randomisation stratified by childcare organisation to achieve balance in the number of preschools within each organisation that will receive the intervention or not (allocation ration 1:1). As preschools within childcare organisations differ in location size (and thus in the number of eligible participants), we will subsequently 1) order preschools by location size, 2) match preschools in pairs and 3) randomly allocate one preschool to the intervention group and the other to the control group using computer-generated randomisation lists.

### Procedures and outcome measures

ECEC teachers will be asked to fill in a questionnaire and participate in a short interview at both baseline and 6 months. Furthermore, parents will be asked to fill in a questionnaire at baseline and at 6 months. The questionnaire for parents will be provided in Dutch, English, Turkish and phonetic Arabic. Around the same time, children’s height, weight and waist circumference will be measured at preschools. Parents in the intervention group will be asked to participate in a short interview about their experience with the toolkit. In addition, at baseline and at 6 months, parents will be invited to participate in a 24 hour recall session about the dietary intake of their child and/or a physical activity measurement of their child.

All data will be coded to protect the privacy of the participants. Data from paper questionnaires and measurement score forms will be handled in Microsoft Excel by double data entry. Interviews will be transcribed non-verbatim and handled in MAXQDA. The qualitative data will be thematically analysed and coded using the 6 phases proposed by Braun and Clarke [24].

The study team will be coordinated by 4 unblinded researchers (SM/NT/MG/MDAVV) and 1 blinded researcher (MTS). MTS will be responsible for final data analyses. Data will be available through the University of Amsterdam / Amsterdam University of Applied Sciences repository called Figshare.

### Socio-demographic characteristics

General socio-demographic data will be obtained via questionnaires for ECEC teachers and parents. ECEC teachers’ age (in years), country of birth, their mother’s and father’s country of birth and highest level of education will be collected. Children’s age (in months), sex, country of birth and their mother’s and father’s country of birth will be collected. In addition, further information about the parents will be collected: their relationship to the child (mother, father or else), their own mother’s and father’s country of birth and the years of education they have had after primary school. The countries of birth will be used to identify a migration background [25,26]. First and second generation migration backgrounds will be taken into account and included as ethnicity (Dutch, Moroccan, Turkish, other western or other non-western) in statistical models. The ECEC teachers’ levels of education will be subdivided in intermediate or higher education groups [27]. The parents’ years of education after primary school will be subdivided in lower, intermediate or higher education levels and will be used as a proxy for socioeconomic position [28].

### ECEC teacher-parent partnership

The ECEC teacher-parent partnership will be examined via questionnaires for ECEC teachers and parents. Furthermore, short interviews with ECEC teachers of about 15 minutes will be held. These semi-structured interviews are intended to give more information about the ECEC teacher-parent partnership regarding eating, physical (in)activity and sleeping behaviours in young children. For example, a researcher will ask when and how ECEC teachers have contact with parents about the 3 lifestyle themes.

Questions in the questionnaire for ECEC teachers are based on the Dutch ‘Monitor Parental Engagement in ECEC settings 2018–2019 for professionals’ [29] and the ‘Family-Centered Practices Scale’ (Short Form) by Dunst and Trivette [30]. The original Short Form by Dunst and Trivette includes a list of 8 statements that describe different ways professionals might interact with and treat families. To make the questions suitable for ECEC teachers to answer and minimise the chance of socially desirable answers, the research staff reformulated the original statements into self-efficacy statements beginning with ‘I feel able to…’. Also, the research staff adjusted the questions to relate them to the lifestyle themes. An additional question was added to the questionnaire for ECEC teachers (‘Do you know where to find information about healthy eating, physical activity and sleeping behaviours for children?’).

Questions in the questionnaire for parents are based on the Dutch ‘Monitor Parental Engagement in ECEC settings 2018–2019 for parents’ [29] and again the ‘Family-Centered Practices Scale’ (Short Form) by Dunst and Trivette [30]. The research staff adjusted the questions to relate them to the lifestyle themes.

Both the questionnaire for ECEC teachers and parents were checked on language level B1 by a member of the Dutch national centre of expertise on health disparities Pharos. Original questions were reformulated to make the questionnaires more accessible to the target population. Answering options for the questions in both questionnaires are never (1 point), very little (2 points), some of the time (3 points), most of the time (4 points) and all the time (5 points) or similar terms. For the questions based on the Dutch ‘Monitor Parental Engagement in ECEC settings 2018–2019’, a mean score per question will be calculated. For the questions based on the ‘Family-Centered Practices Scale’ (Short Form), a sum score per sub scale will be calculated. The ‘Family-Centered Practices Scale’ (Short Form) includes a sub scale for relational family support practices and participatory family support practices.

### ECEC teachers’ and parents’ knowledge, attitude and food- and activity-related practices

Knowledge, attitudes and food- and activity-related practices will be assessed by questionnaires for ECEC teachers and parents.

For ECEC teachers 4 knowledge questions are included. One question was based on the ‘Food and Health Survey 2018’ (‘How familiar are you with the Dutch dietary guidelines for 1–4 year old children?’) [31] and will be separately analysed (answering options: I know nothing about it (1 point), I know a little about it (2 points), I know a fair amount about it (3 points) and I know a lot about it (4 points). The other 3 questions were compiled by the research staff and concern knowledge of the topics healthy eating, physical activity and sleeping behaviours in young children. For parents, only the 3 questions about the lifestyle topics were included. Each correctly answered option will yield 1 point.

Attitudes will be evaluated through individual statements. For ECEC teachers, 4 attitude statements per lifestyle theme (in total 12 questions) are compiled by the research staff. A 5 point Likert-scale will be used. Answering options for the statements are 1) totally disagree (1 point), slightly disagree (2 points), neutral (3 points), slightly agree (4 points) and totally agree (5 points). For parents, the research staff choose to use only 1 attitude question per lifestyle theme (in total 3 questions), as it was intended to make the questionnaire as short as possible to increase accessibility. Parents will be asked to indicate on a scale of 1 (not important at all) to 10 (extremely important), how important they think 1) healthy eating, 2) physical activity and 3) enough sleep is for their child.

A modified version of the ‘Child-care Food and Activity Practices Questionnaire’ (CFAPQ) will be used to assess food- and activity-related practices of ECEC teachers. The original CFAPQ consists of 12 CFAPQ scales (7 food-related and 5 activity-related scales). Gubbels et al. show a sufficient internal consistency in the CFAPQ scales with a Cronbach’s alpha ranging from 0.53 to 0.96 [32]. As not all scales are applicable for the context of preschools, only the following original CFAPQ scales will be used in the questionnaire for ECEC teachers: food-related emotion regulation/food as reward, food-related modelling/encourage balance and variety, food-related pressure to eat, activity-related modelling, activity-related psychological control, activity-related teaching/autonomy support. In addition, there is a single item about how often ECEC teachers encourage the children to eat healthy foods before unhealthy ones. From the original scale food-related involvement/environment the research staff choose to only include the question regarding involvement ‘I allow the children to help prepare meals (for example, set the table, prepare sandwiches, etc.)’ as all preschools have a healthy food policy and therefore the questions regarding environment are not applicable. From the original scale food-related teaching about nutrition also only 1 question will be used (‘I discuss with the children why it’s important to eat healthy foods’) as the original scale showed to have a low Cronbach’s alpha in our previous study at urban preschools [19]. Answering options for the questions are 1) totally disagree (1 point), slightly disagree (2 points), neutral (3 points), slightly agree (4 points), totally agree (5 points) or 2) never (1 point), rarely (2 points), sometimes (3 points), mostly (4 points) and always (5 points). A mean score per scale will be calculated.

### Children’s eating, physical (in)activity and sleeping behaviours

The questionnaire for parents includes questions to obtain data on the eating, physical (in)activity and sleeping behaviours of children. These questions are based on the ‘Sarphati Amsterdam Core set plus questionnaire for 36 months’ [33].

### Dietary intake

To obtain more information about children’s dietary intake, parents will be asked to participate in a multiple-pass 24-hour dietary recall at baseline and at 6 months [34]. Trained students of the AUAS (faculty of Sports and Nutrition) will perform the dietary recall sessions. The sessions will be recorded with Olympus Digital Voice recorders and processed in Microsoft Excel. Foods (g/day) will be classified into groups using the Dutch Food Composition Database 2016 [35].

### Physical activity

Physical activity will be assessed using the 3-axis accelerometer ActiGraph wGT3X-BT. The accelerometers will be attached to an elastic belt on the right hip. Children will wear the accelerometers for 7 consecutive days during waking hours, excluding activities such as bathing and swimming. Data will be derived using a 15-s epoch. A minimum wear time of 6 hours per day will be used for a day to be included in final analysis [36]. Counts per minute will be extracted and used as a measure of physical activity participation in the children [37,38]. Cut-off points described by Trost et al. (2012) will be used to categorise physical activity intensity [39].

### Height, weight and waist circumference

Anthropometric measures of children will be assessed at preschools. Height (cm) will be measured without children’s shoes to the nearest 0.1 cm in standing position using a portable stadiometer (seca 213). Weight (kg) will be measured to the nearest 0.1 kg in standing position without children’s shoes or heavy clothing using a portable weighing scale (seca 813). The measurements will be performed (at least) twice. BMI (kg/m^2^) will be calculated with the mean of the height and weight measurements and BMI z-scores will be assessed using World Health Organisation reference data (WHO Anthro). The weight status of children will be evaluated by reference data of Cole et al. (2012) [40]. Waist circumference (cm) will be measured (at least) twice to the nearest 0.1 cm in standing position at the mid-point between the lower costal margin and the level of the anterior superior iliac using an ergonomic circumference measuring tape (seca 201) [41]. All procedures will be carried out during preschool hours by trained research staff and students of the AUAS. Standard Operation Procedures of the Amsterdam Nutritional Assessment Center of the AUAS will be used.

### Sample size

We aim to include 160 ECEC teachers (n = 80 in the intervention group and n = 80 in the control group). This sample size for ECEC teachers is based on a medium effect size (Cohen’s d: 0.50), a 2-sided alpha of 5%, a power of 80%, a design effect [mean cluster size: 3 (as there are multiple ECEC teachers working at 1 preschool), ICC_teachers_: 0.05] and a 15% drop-out rate. Furthermore, we aim to include 320 parents (mother, father or other primary caregiver) and 320 children (n = 160 parents and their 160 children in the intervention group and n = 160 parents and their 160 children in the control group). Considering a 2-sided alpha of 5% and a power of 80%, it is possible to test with 160 parents and children per group a small to medium effect size (Cohen’s d: 0.35) [mean cluster size: 6, ICC_parents_: 0.02, 15% drop-out rate].

### Statistical analyses

Descriptive statistics n (%) or mean (SD) will be used to describe the characteristics of the study population.

Linear mixed model analyses will be performed to determine the effects of the preschool-based intervention on the primary and secondary outcome measures (intention-to-treat and per protocol analyses). Dummy variables for the childcare organisations will be used and preschool location will be added as a random intercept to take into account the clustered data structure. Overall models with treatment (intervention or control group) and the baseline value for each specific outcome measure will be created to assess the overall treatment effect of the intervention. In addition, the models will be adjusted for age, ethnicity and level of education. Regression coefficients (β), 95% confidence intervals (CI), and P values will be computed for all crude and adjusted mixed models. The statistical significance will be set at p<0.05. With sensitivity analysis, we can explore whether intervention outcomes differ between different subgroups in for example ethnicity and level of education.

## Discussion

This study aims to evaluate the effects of a preschool-based intervention for ECEC teachers and parents with young children. It is hypothesised that the toolkit and associated training for ECEC teachers will improve the ECEC teacher-parent partnership related to healthy eating, physical (in)activity and sleeping behaviours in young children compared to the control group.

Strengths include the randomised controlled study design and a diverse study population to explore generalisation across subgroups. With the extensive recruitment strategy we hope to include a diverse group of ECEC teachers, parents and their children. The intervention materials were produced in close collaboration with our planning group and the materials will be optimised along the way. Intervention materials are simple and culturally sensitive images are used to reach families with different sociocultural and socioeconomic backgrounds. We will obtain, both, teacher- and parent-reported data. A limitation is the use of questionnaires to answer the primary research question (prone to socially desirable answers but the only feasible option in this study). However, we will perform short interviews to also obtain qualitative data on the ECEC teacher-parent partnership regarding eating, physical (in)activity and sleeping behaviours of young children. Furthermore, we planned to objectively measure physical activity with accelerometers.

With this study we will gain important knowledge about the challenging task to engage a diverse group of parents in lifestyle-related topics. The results of our study may inform future studies that aim to design, implement and/or evaluate an aligned intervention for both ECEC teachers and parents. We will provide a practical tool for ECEC teachers to promote a supportive ECEC teacher-parent partnership regarding healthy behaviours in young children. With an improved partnership we intend to contribute to healthy behaviours in disadvantaged young children and minimise health inequalities.

## Supporting information

S1 File SPIRIT checklist. (DOC)

S2 File Protocol. (PDF)
