# Supplementary material for: Effect and process evaluation of a preschool-based intervention to promote an early childhood education and care teacher-parent partnership about healthy behaviours in children: Study protocol for the cluster randomised controlled trial CO-HEALTHY
Source: PLoS One. 2023 Feb 22;18(2):e0281999. doi: 10.1371/journal.pone.0281999 (PMC9946211; doi:10.1371/journal.pone.0281999)
Supplement: S2 File — (PDF) [file pone.0281999.s002.pdf]

RESEARCH PROTOCOL

PROTOCOL TITLE

English: **CO-HEALTHY** - **COll**aboration between parents and childcare to promote a **HEALTHY** lifestyle in young children.

Dutch: **OK-GEZOND** - **O**uderbetrokkenheid op de **K**inderopvang ter bevordering van een **GEZOND**e leefstijl van peuters.

**Protocol ID**	**CO-HEALTHY**
**Short title**	**CO-HEALTHY**
**EudraCT number**	***Not applicable***
**Version**	**4**
**Date**	**12-05-2022**
**Coordinating investigator/project leader**	***Dr. Ir. Martinette T. Streppel***<br>***Faculty of Sports and Nutrition***<br>***Center of Expertise Urban Vitality***<br>***Amsterdam University of Applied Sciences***<br>***Dr. Meurerlaan 8 / 1067 SM Amsterdam***<br>***m.t.streppel@hva.nl***
**Principal investigator(s) (in Dutch: hoofdonderzoeker/ uitvoerder)**	***Prof. Dr. Ir. Peter J.M. Weijs***<br>***Faculty of Sports and Nutrition***<br>***Center of Expertise Urban Vitality***<br>***Amsterdam University of Applied Sciences***<br>***Dr. Meurerlaan 8 / 1067 SM Amsterdam***<br>***p.j.m.weijs@hva.nl***<br><br>***Amsterdam UMC, Location VUmc***<br>***Boelelaan 1117, ZH4A18 / 1081 HV Amsterdam***<br>***p.weijs@amsterdamumc.nl***

**Sponsor (in Dutch: verrichter/opdrachtgever)**	***Amsterdam University of Applied Sciences (AUAS)***
**Subsidising party**	***Nationaal Regieorgaan Praktijkgericht Onderzoek SIA***

TABLE OF CONTENTS

1. INTRODUCTION AND RATIONALE 9
2. OBJECTIVES 10
3. STUDY DESIGN 11
4. STUDY POPULATION 12
4.1 Population (base) 12
4.2 Inclusion criteria 12
4.3 Exclusion criteria 12
4.4 Sample size calculation 12
5. TREATMENT OF SUBJECTS 13
5.1 Investigational product/treatment 13
6. METHODS 19
6.1 Study parameters/endpoints 19
6.1.1 Main study parameter/endpoint 19
6.1.2 Secondary study parameters/endpoints 19
6.1.3 Other study parameters 20
6.2 Randomisation, blinding and treatment allocation 21
6.3 Study procedures 22
6.4 Withdrawal of individual subjects 25
6.5 Replacement of individual subjects after withdrawal 25
6.6 Follow-up of subjects withdrawn from treatment 25
6.7 Premature termination of the study 25
7. SAFETY REPORTING 26
7.1 Temporary halt for reasons of subject safety 26
7.2 AEs and SAEs 26
7.2.1 Adverse events (AEs) 26
7.2.2 Serious adverse events (SAEs) 26
7.3 Follow-up of adverse events 27
8. STATISTICAL ANALYSIS 27
8.1 Primary study parameter(s) 27
8.2 Secondary study parameter(s) 27
8.3 Other study parameters 28
9. ETHICAL CONSIDERATIONS 29
9.1 Regulation statement 29
9.2 Recruitment and consent 29
9.3 Objection by minors or incapacitated subjects 29
9.4 Benefits and risks assessment, group relatedness 30
9.5 Compensation for injury 30
9.6 Incentives 30
10. ADMINISTRATIVE ASPECTS, MONITORING AND PUBLICATION 31
10.1 Handling and storage of data and documents 31
10.2 Amendments 32
10.3 Annual progress report 32

10.4 Temporary halt and (prematurely) end of study report 32
10.5 Public disclosure and publication policy 33
11. STRUCTURED RISK ANALYSIS 34
11.1 Potential issues of concern 34
11.2 Synthesis 34
12. REFERENCES 35

LIST OF ABBREVIATIONS AND RELEVANT DEFINITIONS

ABR	General Assessment and Registration form (ABR form), the application form that is required for submission to the accredited Ethics Committee; in Dutch: Algemeen Beoordelings- en Registratieformulier (ABR-formulier)
AE	Adverse Event
AR	Adverse Reaction
AUAS	Amsterdam University of Applied Sciences
BMI	Body Mass Index
CA	Competent Authority
CCMO	Central Committee on Research Involving Human Subjects; in Dutch: Centrale Commissie Mensgebonden Onderzoek
CV	Curriculum Vitae
ECEC	Early Childhood Education and Care
EU	European Union
EudraCT	European drug regulatory affairs Clinical Trials
GCP	Good Clinical Practice
GDPR	General Data Protection Regulation; in Dutch: Algemene Verordening Gegevensbescherming (AVG)
IB	Investigator’s Brochure
IC	Informed Consent
METC	Medical research ethics committee (MREC); in Dutch: medisch-ethische toetsingscommissie (METC)
RCT	Randomised Controlled Trial
(S)AE	(Serious) Adverse Event
Sponsor	The sponsor is the party that commissions the organisation or performance of the research, for example a pharmaceutical company, academic hospital, scientific organisation or investigator. A party that provides funding for a study but does not commission it is not regarded as the sponsor, but referred to as a subsidising party.
SUSAR	Suspected Unexpected Serious Adverse Reaction
UAVG	Dutch Act on Implementation of the General Data Protection Regulation; in Dutch: Uitvoeringswet AVG
WMO	Medical Research Involving Human Subjects Act; in Dutch: Wet Medisch-wetenschappelijk Onderzoek met Mensen

SUMMARY

Rationale: The need for early interventions to prevent overweight and obesity in deprived areas is widely recognised. Urban preschools with play-based education provide an opportunity to reach many young children (2 to 4 years old) and their parents with disadvantaged backgrounds. Early Childhood Education and Care (ECEC) teachers at preschools are potential key figures to promote healthy behaviours and engage parents in lifestyle-related topics. A partnership between ECEC teachers and parents regarding eating, physical (in)activity and sleeping behaviours may support parents and stimulate their children's development. However, it is not an easy task to establish such a collaboration in practice.

Objective: To examine the effects of a preschool-based intervention on the ECEC teacher-parent partnership regarding eating, physical (in)activity and sleeping behaviours in children.

Study design: Cluster Randomised Controlled Trial.

Study population: ECEC teachers, parents and their children (2 to 4 years old) at urban preschools in Amsterdam, the Netherlands, that provide play-based education.

Intervention: The intervention consists of a training and a toolkit for ECEC teachers. The Toolkit CO-HEALTHY includes 10 parent-child activities concerning healthy eating, physical (in)activity and sleeping behaviours in young children. At intervention preschools, a local team of ECEC teachers will select 6 parent-child activities (most suitable for their group of parents/children) and will carry out the activities during regular contact moments. Parents will receive associated intervention materials and will be encouraged to perform parent-child activities at home. ECEC teachers at control preschools will not receive the training and Toolkit.

Main study parameters/endpoints: The ECEC teacher-parent partnership regarding eating, physical (in)activity and sleeping behaviours in young children, which will be assessed by questionnaire at baseline and at 6 months after the start of the intervention.

Nature and extent of the burden and risks associated with participation, benefit and group relatedness: The risks associated to the intervention are minimal. ECEC teachers are used to provide parent-child activities. In this study, 6 parent-child activities about eating, physical (in)activity and sleeping behaviours of young children will be carried out and supported by local teams of ECEC teachers during regular contact moments at preschools. ECEC teachers will be asked to fill in a questionnaire and participate in a short interview at both baseline and 6 months. Parents will be asked to fill in a questionnaire at baseline and at 6 months. At 6 months, only parents in the intervention group will additionally be asked to participate in a short interview about the intervention. Children's height, weight and waist circumference will be measured at baseline and at 6 months. In addition, at baseline and at 6 months, parents will be given the option to participate in a 24 hour recall about the dietary intake of their child and/or a physical activity measurement of their child (accelerometer for 7

consecutive days). Benefits include the free of charge training and Toolkit for ECEC teachers and healthy life style activities for parents/children.

1. INTRODUCTION AND RATIONALE

The problem of overweight and obesity in children is more severe in deprived urban settings, where it is related to the relatively high number of families with a migration background and/or low socio-economic status (1). Children show health inequalities already in the preschool period, and the inequalities widen with increasing age (2-4). Overweight or obese children have a higher risk of developing non-communicable diseases and psychosocial impairments earlier in life (5). Furthermore, they are more likely to become overweight or obese adults (6). The period between age 2 and 6 is described to be important for the development of adult overweight (7). These findings emphasise the need for early interventions to prevent excess weight gain in young children, especially in deprived areas to minimise health inequalities.

In the Netherlands, urban preschools provide an excellent opportunity to reach young children (2 to 4 years old) from families with a migration background and/or low socio-economic status. In particular, parents of children at risk for language or developmental delays are advised to enrol their child in these community-based services in deprived areas. The urban preschools provide play-based education and prepare children for primary school. Children generally spend up to 16 hours per week in preschool and these settings are therefore important environments for early interventions in children with disadvantaged backgrounds (8, 9).

Early Childhood Education and Care (ECEC) teachers at urban preschools are potential key actors in interventions to prevent overweight and obesity in children. The teachers may promote healthy behaviours at preschools and engage parents in lifestyle-related topics. Parental engagement is suggested to be essential for early interventions to be successful. Ward et al. (2017), conclude in their systematic review that there is tentative evidence that multi-component and multi-level ECEC-based interventions with parental engagement are most likely to have an effect on children’s weight development (10). Furthermore, van der Kolk et al. (2019) report in a recent systematic review that interventions in ECEC settings with direct parental involvement show promising effects on health-related behaviours. However, the authors emphasise that evidence is limited, in particular for anthropometric outcomes (11).

Engaging parents to stimulate healthy behaviours in children is not an easy task. In the context of promoting a healthy lifestyle in young children, even a partnership between ECEC teachers and parents is desired as transfer from the ECEC center to the families’ homes is the intended outcome. Dev et al. (2017), conducted a qualitative study on perspectives of ECEC teachers in communicating with parents about nutrition and health. Barriers to engage parents include: parents are too busy, teachers are unsure of how to communicate about nutrition without offending parents, and teachers are concerned if parents are receptive to nutrition education materials. Reported successful strategies for communication include: recognising the benefits of communicating with parents about nutrition to support child health and building a partnership with parents through parent education (12). In practice, ECEC teachers indicate that they need

tools to communicate about healthy lifestyle-related topics and start building a partnership with parents (13).

In this study CO-HEALTHY, the effects of a training and toolkit for ECEC teachers will be examined. The training focusses on improving teachers' knowledge and skills to collaborate with parents regarding healthy behaviours of their children. The Toolkit includes 10 parent-child activities concerning healthy eating, physical (in)activity and sleeping behaviours in young children. Together, the training and Toolkit will provide a practical guide for ECEC teachers at urban preschools to communicate with parents about lifestyle-related topics. Local teams of ECEC teachers will select 6 parent-child activities (most suitable for their group of parents/children) from the Toolkit and will carry out the activities during regular contact moments. Parents will receive associated intervention materials and will be encouraged also to perform parent-child activities at home. It is aimed to improve the partnership between ECEC teachers and parents regarding lifestyle behaviours of their children. The intervention should reach parents and their children with different socio-economic and socio-cultural backgrounds.

2. OBJECTIVES

Primary Objective

To examine the effects of the preschool-based intervention CO-HEALTHY on the **ECEC teacher-parent partnership** regarding eating, physical (in)activity and sleeping behaviours in young children (2 to 4 years old).

Secondary Objectives

To examine the effects of the preschool-based intervention CO-HEALTHY on:

- ECEC teachers' knowledge, attitude and practices regarding eating, physical (in)activity and sleeping behaviours in young children;
- parents' knowledge, attitude and practices regarding eating, physical (in)activity and sleeping behaviours in young children;
- children's eating, physical (in)activity and sleeping behaviours;
- children's dietary intake;
- children's physical activity level;
- children's height;
- children's weight;
- children's waist circumference;
- children's BMI (z-score).

3. STUDY DESIGN

The present study concerns a cluster Randomised Controlled Trial (cluster-RCT) to examine the effects of the preschool-based intervention CO-HEALTHY. Per participating childcare organisation, the urban preschools (clusters) will be randomly allocated to an intervention or control group. The study period is 6 months. Data of ECEC teachers, parents and children will be collected at baseline and at 6 months. Figure 1 shows a schematic overview of the study.

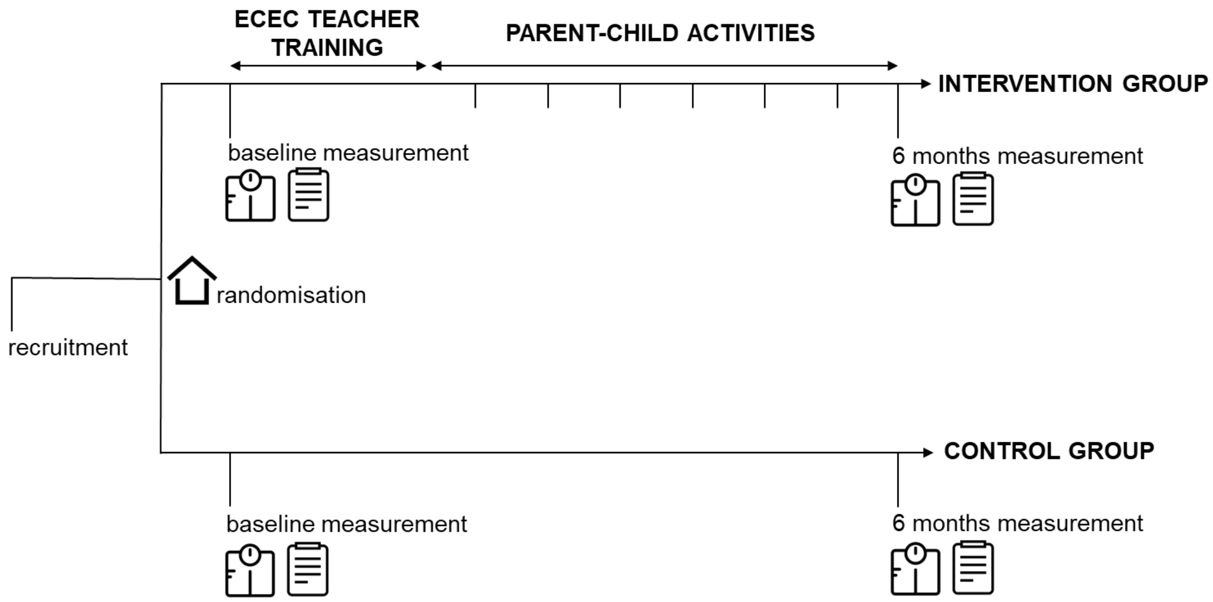

Figure 1. Schematic overview of the study

4. STUDY POPULATION

4.1 Population (base)

The study population will consist of ECEC teachers, parents and their children at urban preschools in Amsterdam. Childcare organisations ‘Impuls Kinderopvang’, ‘Smallsteps Kinderopvang / KidsFoundation’ and ‘Swazoom’ are involved in this study. The participating organisations have urban preschools, mainly located in Amsterdam Nieuw-West and Zuidoost. These city districts are characterised by a relatively high level of inhabitants with a migration background and/or low socio-economic status (14). In 2017, 12.9% and 9.9% of children aged 3 years were reported to be overweight or obese in Amsterdam Nieuw-West and Zuidoost, respectively (15).

All ECEC teachers of the participating preschools will be approached to participate in the study. Parents will be individually approached at preschools and will be asked to provide consent for the participation of their child.

4.2 Inclusion criteria

- Urban preschools must provide play-based education to be eligible to be included in this study.
- ECEC teachers and parents must provide written Informed Consent for participation.
- Parents must provide additional written Informed Consent for the participation of their child.
- Parents must have a child at the participating urban preschools and the child must be between 2 to 3.5 years old to be eligible to participate in this study.

4.3 Exclusion criteria

- ECEC interns will be excluded from participation of this study.

4.4 Sample size calculation

It is aimed to include 160 ECEC teachers (n = 80 in the intervention group and n = 80 in the control group). This sample size is based on a medium effect size (Cohen’s d: 0.50), a 2-sided alpha of 5%, a power of 80%, a design effect (mean cluster size: 3, ICC: 0.05) and a 15% drop-out rate. Furthermore, it is aimed to include 320 parents and 320 children (n = 160 parents and their 160 children in the intervention group and n = 160 parents and their 160 children in the control group). Considering a 2-sided alpha of 5% and a power of 80%, it is possible to obtain, with 160 parents and children per group, a small to medium effect size (Cohen’s d: 0.35) (mean cluster size: 6, ICC: 0.02, 15% drop-out rate).
Egert et al. (2020), conducted a meta-analysis and showed a small to medium effect of professional development in ECEC on the quality of teacher-child interactions (16).

5. TREATMENT OF SUBJECTS

5.1 Investigational product/treatment

At intervention preschools, at least 1 ECEC teacher will follow a 3-hour training session. The training focusses on improving the teachers' knowledge and skills to collaborate with parents regarding lifestyle behaviours of their children. The first part of the training will include theory and a practical assignment about collaborating with parents. In the second part of the training, the Toolkit with 10 parent-child activities concerning eating, physical (in)activity and sleeping behaviours in young children will be presented. The parent-child activities aim to engage parents from different socio-economic and socio-cultural backgrounds. ECEC teachers will be instructed to carry out 6 of the 10 parent-child activities in a period of 3 months. A reader with information from the training will be provided. After the training session, the ECEC teachers will communicate the content of the training to their local team of colleague ECEC teachers in about 1 hour. As a team, the ECEC teachers will decide which parent-child activities they will provide at their preschool. The parent-child activities, of about 15 minutes each, will be carried out at regular contact moments during preschool hours (at the walk-in play time/when parents bring their child). Parents will receive corresponding intervention materials per activity and will be encouraged also to perform parent-child activities at home. Because of the corona measures, the walk-in play time does not exist as parents are not allowed to enter the preschools. The researchers changed the initial parent-child activities slightly in a matter that the activities can, in times of corona, be carried out by ECEC teachers with children at preschool and by parents with children at home. Please see a short description of the activities in the next section. When parents are allowed to enter the preschools again, the original activities at preschools will be implemented. In the end, 2 versions of the Toolkit will be taken into account in data analyses. The participating childcare organisations provided consent to train ECEC teachers and to carry out the parent child activities at intervention preschools. ECEC teachers at control preschools will not receive the training and Toolkit.

PARENT-CHILD ACTIVITIES

Activity 1 - breakfast

Parents and children select pictures of healthy food products for breakfast.

Parents will receive a folder with plasticised pictures of healthy food products for breakfast and a placemat with an image of a child's plate/cup. Children and parents select pictures of food products they like and stick the selected pictures on the plate/cup (via hook-and-loop fasteners).

Next, ECEC teachers will show an information card with practical tips for providing a healthy breakfast at home. Parents will receive a copy of the information card and will be asked to organise a healthy breakfast at home in the upcoming week.

Alternative activity in times of corona

Parents will receive the information card with practical tips for providing a healthy breakfast at home. In addition, parents will be given a placemat with an image of a child’s plate/cup and A4 with pictures of healthy food products for breakfast. Parents can cut/tear the products together with their child and let their child choose a tasty breakfast. Next, they can glue/paste the chosen products on the placemat. The placemats can be brought to the preschool and ECEC teachers will hang (together with the children) the placemats in the classroom. Parents will be asked to organise a healthy breakfast at home in the upcoming week.

Activity 2 - eating vegetables and helping in the kitchen

Parents and children prepare fruit and vegetables.

Parents will receive a cutting board, plastic knife (for the children) and a regular knife (for the parents). Children and parents wash their hands, wash the fruits/vegetables and cut the fruits/vegetables together.

Next, ECEC teachers will show an information card with practical tips about eating vegetables and helping in the kitchen. Parents will receive a copy of the information card to take home. On the back of the information card children can put a sticker when they taste a vegetable at home. Parents will be asked to let their child help in the kitchen at home in the upcoming week.

Alternative activity in times of corona

ECEC teachers prepare fruit and vegetables together with the children at preschool. They will tell parents that they performed this activity at preschool. In addition, they will give parents the information card with practical tips about eating vegetables and helping in the kitchen and the plastic knife to take home. On the back of the information card children can put a sticker when they taste a vegetable at home. Parents will be asked to let their child help in the kitchen at home in the upcoming week.

Activity 3 - healthy eating

Parents and children play a memory game.

Parents will receive a memory game and will be asked to play the game together with their child. When the child finds 2 memory cards that belong together, they can hang the cards

with clothespins to a clothesline in the classroom. Next, ECEC teachers will show an information card with practical tips about healthy eating. Parents will receive a copy of the information card to take home. The back of the information card shows healthy snacks for children. Parents will be asked to let their child choose a healthy snack to try at home in the upcoming week.

Alternative activity in times of corona

Parents will receive the memory game to take home and will be asked to play the game together with their child. In addition, parents will receive the information card with practical tips about healthy eating. The back of the information card shows healthy snacks. Parents will be asked to let their child choose a healthy snack to try at home in the upcoming week.

Activity 4 - portion sizes

Parents and children decorate a placemat.

Parents will receive a placemat and colours. Children colour the placemat together with parents. Next, ECEC teachers show an information card with practical tips about providing healthy portion sizes.

Parents will receive a copy of the information card and the coloured placemat to take home. Children can help setting the table at home by using the placemat.

Alternative activity in times of corona

Children will colour a placemat at preschool. Parents will receive the coloured placemat and an empty one to take home. Children can colour the placemat together with their parents at home and can help setting the table by using the placemats. In addition, parents will receive the information card with practical tips about providing healthy portion sizes.

Activity 5 - drinking water

Parents and children decorate a water cup

Parents will receive a water cup and will be asked to decorate the cup together with their child. In the classroom there is a lemonade tap with water (and pieces of fruit). Children can tap water with their decorated cups. Next, ECEC teachers will show an information card with practical tips about drinking water and a poster about the amount of sugar in several sweetened beverages. Parents will receive a copy of the information card. On the back of the information card children can put a sticker when they drink water at home.

Alternative activity in times of corona

Children will decorate a water cup at preschool together with the ECEC teachers. In the classroom there is a lemonade tap with water (and pieces of fruit). Children can tap water with their decorated cups. Parents will receive the decorated cup and the information card with practical tips about drinking water to take home. On the back of the information card children can put a sticker when they drink water at home.

Activity 6 - do a physical activity together

Parents and children carry out a physical activity together.

A physical activity calendar with a parent-child activity for each month of the year will hang in the classroom. Children and parents perform 1 physical activity of the calendar at preschool. This activity is balancing on a line. Balance lines (zigzag, straight and corners) are drawn on to the floor with painter's tape. Children can walk forward, sideways and backwards on the lines with parents. An optional extra challenge is walking on toes or 1 leg. ECEC teachers can add roadblocks/obstacles to step over.

Next, parents will receive a small version of the calendar and an information card with practical tips about exercising together to take home. Parents will be asked to carry out a physical activity at home with their children in the upcoming week. The physical activity calendar will remain hanging in the classroom, so every month there is a different physical activity at home and at the preschool.

Alternative activity in times of corona

Children will do the activity "balancing on a line" together with the ECEC teachers at preschool. ECEC teachers will tell parents that they did this activity at preschool and will give parents the physical activity calendar and information card with practical tips to exercise together to take home. Parents will be asked to do a physical activity from the calendar together with their child at home in the upcoming week.

Activity 7 - screen time/playing indoors

Parents and children do a search game.

Parents will be given a kitchen timer and will be asked to set the timer to 30 seconds or 1 minute. Next, parents will ask their child to search for a certain object or an object with a specific colour. When the alarm goes off, the children stop searching.

Next, ECEC teachers will show an information card with practical tips playing inside and reducing screen time. Parents will receive a copy of the information card to take home and will be told that rules about screen time can be set at home using a timer (for example, a maximum of 5 minutes screen time at a time).

Alternative activity in times of corona

ECEC teachers will perform the search game together with the children at preschools. The teachers will tell parents that they did this activity at preschool and will give parents a short instruction to play the game at home. In addition, parents will receive the information card with practical tips about playing inside and reducing screen time. They will be told that rules about screen time can be set at home using a timer (for example, a maximum of 5 minutes screen time at a time).

Activity 8 - exercising together

Children practice a balance bike trail together with their parents.

ECEC teachers prepare a trail with chalk (a slalom track/objects to stop at) outside. Children practice riding a balance bike on the trail supported by their parents. ECEC teachers give tips for using the balance bike. In addition, there's another trail (climbing on objects/jumping in circles) to do by foot for the children who have to wait for a bike.

A loan system for balance bikes will be set up. Children can take home a balance bike for a week. Parents receive an information card with practical tips for using the balance bike at home.

Alternative activity in times of corona

Children will practice riding a balance bike on the trail at preschool. ECEC teachers will tell parents that they did this activity at preschool. A loan system for balance bikes will be set up. Children can take home a balance bike for a week. Parents receive the information card with practical tips for using the balance bike at home.

Activity 9 - playing outside

Parents and children look outside for a natural treasure.

Parents and children will be shown a poster with an image of a tree. Children and their parents search for a "natural treasure" outside (for example, beautiful leaves). The treasures will then be glued on to the tree, so children and their parents make art from nature.

Next, ECEC teachers will show an information card with practical tips for playing outside. Parents will receive a copy of the information card and will be asked to play outside with their children every day in the upcoming week. In addition, parents receive a list of (free) exercise places in the neighbourhood.

Alternative activity in times of corona

Children and their parents search for a "natural treasure" outside (for example, beautiful leaves). Children bring the treasures to the preschool and glue them on a poster with an image of a tree, so children make art from nature together with the ECEC teachers. Parents will be asked to play outside with their children every day in the upcoming week. In addition, parents receive the list of (free) exercise places in the neighbourhood and the information card with practical tips for playing outside to take home.

Activity 10 - sleeping

Parents and children create a ritual with sleep ritual cards.

Parents will be given sleep ritual cards (with images such as brushing teeth, bedtime story, tucking in). Children and their parents put the cards in a sequence so that it becomes their own sleep ritual. Children can practice 1 of the activities, shown in the sleep ritual cards, on a doll/cuddly toy in the room.

Next, ECEC teachers will show an information card with practical tips about sleeping. Parents will receive a copy of the information card to take home and will be asked to perform the sleep ritual at home in the upcoming week.

Alternative activity in times of corona

ECEC teachers will ask children what things they can do before they go to bed. The teachers will show all sleep ritual card and discuss them with the children. Children can practice 1 of the activities, shown in the sleep ritual cards, on a doll/cuddly toy in the room. ECEC teachers will tell parents that they did this activity at preschool and will give parents 2 A4 to take home, 1 with pictures of 8 sleep ritual cards and 1 with a plain sequence. Children and their parents can cut/tear the sleep ritual cards and glue them in a sequence on the second A4 so that it becomes their own sleep ritual. In addition, parents will receive the information card with practical tips about sleeping to take home and will be asked to perform the sleep ritual at home in the upcoming week.

6. METHODS

A detailed description of the study procedures and methods to assess the study parameters can be found in section 6.3. Table 1 shows an overview of the study parameters per study timepoint.

6.1 Study parameters/endpoints

6.1.1 Main study parameter/endpoint

Changes in scores on the **ECEC teacher-parent partnership** regarding eating, physical (in)activity and sleeping behaviours in young children. *Assessed by questionnaire at baseline and at 6 months, multiple informed research (teacher informed and parent informed).*

6.1.2 Secondary study parameters/endpoints

ECEC TEACHERS

Changes in scores on the **knowledge, attitude and practices** regarding healthy eating, physical (in)activity and sleeping behaviours of young children. *Assessed by questionnaire at baseline and at 6 months.*

Experiences with the toolkit (only applicable for ECEC teachers in the intervention group). *Obtained with a short interview at 6 months.*

PARENTS

Changes in scores on the **knowledge, attitude and practices** regarding healthy eating, physical (in)activity and sleeping behaviours of young children. *Assessed by questionnaire at baseline and at 6 months*

Whether or not they performed **parent-child activities at home**. If yes, which activities. If not, why not. *Assessed by questionnaire at 6 months,* o*nly applicable for parents in the intervention group.*

Experiences with the toolkit (only applicable for parents in the intervention group). *Obtained with a short interview at 6 months.*

CHILDREN

Eating, physical (in)activity and sleeping behaviours

Assessed by questionnaire at baseline and at 6 months.

In times of corona, researchers and parents are not allowed to enter the preschools. Therefore it is not possible to perform the following measurements at preschools. When it is allowed again to enter preschools, the researchers will start performing the measurements and collect data on children's dietary intake, physical activity levels and weight development.

Dietary intake

Parents will be given the option to participate in a 24 hour recall about the dietary intake of their child at baseline and at 6 months.

Physical activity

Parents will be given the option to participate in a physical activity assessment (accelerometer) of their child at baseline and at 6 months.

Height (cm)

Measurement at baseline and at 6 months.

Weight (kg)

Measurement at baseline and at 6 months.

Waist circumference (cm)

Measurement at baseline and at 6 months.

BMI (z-score)

BMI (kg/m^2) will be calculated and BMI z-scores will be assessed at baseline and at 6 months.

6.1.3 Other study parameters

ECEC TEACHERS

Socio-demographic characteristics (age, sex, ethnicity and educational level).

PARENTS

Socio-demographic characteristics (age, sex, ethnicity and educational level).

CHILDREN

Socio-demographic characteristics (age, sex, ethnicity).

Table 1. Overview of the study parameters per study timepoint

TIMEPOINT	BASELINE			6 MONTHS		
	**Teachers**	**Parents**	**Children**	**Teachers**	**Parents**	**Children**
**Socio-demographic characteristics**	X	X	X			
**Partnership**	X	X		X	X	
**Knowledge**	X	X		X	X	
**Attitude**	X	X		X	X	
**Practices**	X	X		X	X	
**Eating behaviours**			X			X
**Physical (in)activity behaviours**			X			X
**Sleeping behaviours**			X			X
**Dietary intake**			X			X
**Physical (in)activity**			X			X
**Height**			X			X
**Weight**			X			X
**Waist circumference**			X			X
**BMI (z-score)**			X			X

6.2 Randomisation, blinding and treatment allocation

Randomisation will be performed before the start of the study on the level of preschools within participating childcare organisations. The randomisation will be performed by an independent researcher of the AUAS with the use of computer-generated randomisation lists. It concerns a stratified randomisation by childcare organisation to achieve balance in the number of preschools within each organisation that will receive the intervention or not. As preschools within each organisation differ in location size (and therefore the number of eligible participants), we will 1) order preschools by location size, 2) match preschools in pairs based on location size and 3) randomly allocate 1 preschool to the intervention group and the other to the control group. 1 member of the research staff (Martinette T. Streppel) will be blinded for allocation and perform final analyses. ECEC teachers, parents and other members of the research staff will not be blinded as the intervention does not allow for this.

6.3 Study procedures

The study period will be 6 months, starting with a baseline measurement and ending with a close-out measurement. All assessments will take place at preschools in Amsterdam, the Netherlands. In times of corona, only questionnaires will be distributed as other measurements at preschools are not allowed.

ECEC TEACHERS

At baseline and at 6 months, participating ECEC teachers will be provided with a paper questionnaire. In times of corona, a digital version of the questionnaire for ECEC teachers will be used. The questionnaire includes questions to obtain ECEC teachers' age, sex, highest level of education, country of birth and parents' country of birth. The highest level of education (proxy for socio-economic status) of ECEC teachers will be subdivided in intermediate or higher education groups (17). Ethnicity will be based on first- and second-generation migration backgrounds. First-generation migration backgrounds will be defined as the country of birth. Second-generation migration backgrounds will be defined as the mother's country of birth, or, in case the mother's country of birth is the Netherlands, as the father's country of birth (18). The aim of this study is to examine the effects of a preschool-based intervention on the ECEC teacher-parent partnership regarding eating, physical (in)activity and sleeping behaviours in children. Ethnicity could play a role in this partnership. For example, when an ECEC teacher and parent have the same ethnicity, it could improve their partnership. A diverse group of ECEC teachers (and parents, see next paragraph) is expected, because teachers often live in the city district (Amsterdam Nieuw-West or Zuidoost) of their preschool. The city districts Amsterdam Nieuw-West and Zuidoost are known for their ethnic diversity. Ethnicity may occur as a moderator and/or mediator, and is therefore an indispensable variable (19).

Furthermore, the questionnaire for ECEC teachers includes questions on the ECEC teacher-parent partnership regarding eating, physical (in)activity and sleeping behaviours in young children. These questions are based on the Dutch 'Monitor Samenwerken met ouders 2018-2019' (20) and the 'Family-Centered Practices Scale (Extended Version)' by Dunst and Trivette (21). Answering options for the questions are totally disagree (1 point), slightly disagree (2 points), neutral (3 points), slightly agree (4 points), totally agree (5 points) or never (1 point), rarely (2 points), sometimes (3 points), mostly (4 point), always (5 points). For the questions based on the Dutch 'Monitor Samenwerken met ouders 2018-2019', a mean score per question will be calculated. For the questions based on the 'Family-Centered Practices Scale (Extended Version)', a mean score per scale will be calculated. The 'Family-Centered Practices Scale (Extended Version)' includes 2 scales: 1 for relational family support practices and 1 for participatory family support practices. ECEC teachers can use these practices to build a partnership with parents (21).

The questionnaire for ECEC teachers also includes questions to obtain data on the teachers' knowledge, attitude and practices about healthy behaviours in young children. These questions are based on the Food and Health Survey 2018 (22) and the

Child-care Food and Activity Practices Questionnaire (23). In addition, some questions are compiled by the research staff. Per question or scale (applicable for questions on food/activity-related practices) a mean score will be calculated. ECEC teachers can fill in the questionnaire during 'task hours' at preschool (it will take about 30 minutes to fill in the questionnaire). The research staff will visit preschools to collect the questionnaires (1 week after the questionnaires are being provided). At the collection moment, the research staff will ask, in short interviews of about 15 minutes, some additional questions regarding the ECEC teacher-parent partnership. These questions will be based on the teachers' answers in the completed questionnaire. In times of corona, the short interviews with ECEC teachers will be held by telephone.

PARENTS

At baseline and at 6 months, participating parents will be provided with a paper questionnaire (provided in the 4 most spoken languages: phonetic Arabic, Turkish, Dutch and English). The questionnaire includes questions to obtain parents'/children's age, sex, level of education (only applicable for parents, country of birth and parents' country of birth. The highest level of education (proxy for socio-economic status) of parents will be subdivided in lower, intermediate or higher education groups (17). Ethnicity will be based on first- and second-generation migration backgrounds. First-generation migration backgrounds will be defined as the country of birth. Second-generation migration backgrounds will be defined as the mother's country of birth, or, in case the mother's country of birth is the Netherlands, as the father's country of birth (18).The intervention should reach parents and their children with different socio-economic and socio-cultural backgrounds. To investigate if we indeed reach all parents and children it is important to gain insight in the level of education and ethnicity of parents and their children. Moreover, the intervention is intended to improve the ECEC teacher-parent partnership regarding eating, physical (in)activity and sleeping behaviours in children. Ethnicity could play a role in this partnership. For example, when an ECEC teacher and parent have the same ethnicity, it could improve their partnership. A diverse group of parents (and teachers, see previous paragraph) is expected, because parents often live in the city district (Amsterdam Nieuw-West or Zuidoost) of their preschool. The city districts Amsterdam Nieuw-West and Zuidoost are known for their ethnic diversity. Level of education and ethnicity may occur as moderators and/or mediators, and are therefore indispensable variables. It is, therefore, important to obtain information about the above mentioned socio-demographic characteristics. Ethnicity and level of education may occur as moderators and/or mediators, and are therefore needful variables (19).

Furthermore, the questionnaire for parents includes questions on the ECEC teacher-parent partnership regarding eating, physical (in)activity and sleeping behaviours in young children. These questions are, just like in the questionnaire for ECEC teachers, based on the Dutch 'Monitor Samenwerken met ouders 2018-2019' (20) and the 'Family-Centered Practices Scale (Extended Version)' by Dunst and Trivette (21).

Answering options for the questions are the same as in the questionnaire for ECEC teachers and mean scores per question/scale will be calculated.
The questionnaire for parents also includes questions to obtain data on the eating, physical (in)activity and sleeping behaviours of their child. These questions are based on the 'Sarphati Amsterdam Core set plus questionnaire for 36 months' (24). In addition, some questions are included on the parents' knowledge, attitude and practices about healthy behaviours for young children. These questions are based on the Food and Health Survey 2018 (22), Comprehensive Feeding Questionnaire (25), Preschooler Physical Activity Parenting Practices questionnaire (26), and a questionnaire on maternal efficacy (27). In addition, some questions are compiled by the research staff. Parents can fill in the questionnaire at home (it will take about 30 minutes to fill in the questionnaire). The research staff will visit preschools to collect the questionnaires (1 week after the questionnaires are being provided). To obtain more information about children's dietary intake, parents will be asked to participate in a 24-hour dietary recall at baseline and at 6 months. In times of corona, 24-hour dietary recall sessions will not be performed as parents and researchers are not allowed to enter the preschools. When it is allowed again to enter preschools, the researchers will start to collect data on children's dietary intake. Paper questionnaires will, in times of corona, be distributed (at the door) by ECEC teachers. To obtain input for a process evaluation of the intervention, short interviews with participating parents will be held at 6 months (only applicable for parents in the intervention group). At 6 months, the research staff will ask parents if they would like to participate in a short interview of about 15 minutes and give their opinion about the parent-child activities. If yes, the research staff will note a telephone number and will call the specific parent for the short interview concerning the intervention.

CHILDREN
At baseline and 6 months, children's height, weight and waist circumference will be measured at preschools. In addition, (some) children will wear an accelerometer for 7 consecutive days. Height (cm) will be measured to the nearest 0.1 cm in standing position using a portable stadiometer (Seca 213). Weight (kg) will be measured to the nearest 0.1 kg in standing position without shoes or heavy clothing using a portable weighing scale (Seca 813). BMI (kg/m^2) will be calculated and BMI z-scores will be assessed using World Health Organisation reference data (WHO Anthro). Waist circumference (cm) will be measured to the nearest 0.1 cm in standing position (at the mid-point between the lower costal margin and the level of the anterior superior iliac) with a standard tape measure (28). All procedures will be performed using Standard Operating Procedures by trained research staff/students of the AUAS. Physical activity will be assessed using a 3-axis accelerometer: ActiGraph GT3X+ (29). The accelerometers will be placed on the right hip using an elastic belt. Children will wear the accelerometers for 7 consecutive days during waking hours, excluding activities including water such as bathing/showering and swimming. Data will be derived using a 10-s epoch. Counts per minute based on vector magnitude will be extracted. In times

of corona, physical measurements at preschools will not be performed as parents and researchers are not allowed to enter the preschools. When it is allowed again to enter preschools, the researchers will start performing the measurements and collect data on children's weight development and physical activity levels.

6.4 Withdrawal of individual subjects

Participants can leave the study at any time for any reason if they wish to do so without any consequences. Parents can end the child's participation at any time for any reason if they wish to do so without any consequences. If a child expresses an objection in the measurements, the measurements will be discontinued.

6.5 Replacement of individual subjects after withdrawal

Participants will not be replaced after withdrawal.

6.6 Follow-up of subjects withdrawn from treatment

There will be no follow-up of patients who withdraw from the study.

6.7 Premature termination of the study

There are no reasons available for discontinuation of this study.

7. SAFETY REPORTING

7.1 Temporary halt for reasons of subject safety

In accordance to section 10, subsection 4, of the WMO, the sponsor will suspend the study if there is sufficient ground that continuation of the study will jeopardise subject health or safety. The sponsor will notify the accredited METC without undue delay of a temporary halt including the reason for such an action. The study will be suspended pending a further positive decision by the accredited METC. The investigator will take care that all subjects are kept informed.

7.2 AEs and SAEs

7.2.1 Adverse events (AEs)

Adverse events are defined as any undesirable experience occurring to a subject during the study, whether or not considered related to the experimental intervention. All adverse events reported spontaneously by the subject or observed by the investigator or his staff will be recorded.

7.2.2 Serious adverse events (SAEs)

A serious adverse event is any untoward medical occurrence or effect that:

- results in death;
- is life threatening (at the time of the event);
- requires hospitalisation or prolongation of existing inpatients' hospitalisation;
- results in persistent or significant disability or incapacity;
- is a congenital anomaly or birth defect; or
- any other important medical event that did not result in any of the outcomes listed above due to medical or surgical intervention but could have been based upon appropriate judgement by the investigator.

An elective hospital admission will not be considered as a serious adverse event.

The investigator will report all SAEs to the sponsor without undue delay after obtaining knowledge of the events, except for the following SAE:

- an elective hospital admission.

The sponsor will report the SAEs through the web portal *ToetsingOnline* to the accredited METC that approved the protocol, within 7 days of first knowledge for SAEs that result in death or are life threatening followed by a period of maximum of 8 days to complete the initial preliminary report. All other SAEs will be reported within a period of maximum 15 days after the sponsor has first knowledge of the serious adverse events.

7.3 Follow-up of adverse events

All AEs will be followed until they have abated, or until a stable situation has been reached. Depending on the event, follow up may require additional tests or medical procedures as indicated, and/or referral to the general physician or a medical specialist.

SAEs need to be reported till end of study within the Netherlands, as defined in the protocol.

8. STATISTICAL ANALYSIS

Descriptive statistics (n (%) or mean ± standard deviation) will be used to describe the characteristics of the study population.

Linear mixed model analyses will be performed to determine the effects of the preschool-based intervention on the primary and secondary outcome measures. Childcare organisation and preschool location will be added as random intercepts to take into account the clustered data structure. Overall models with treatment (intervention or control group) and the baseline value of a specific outcome measure will be made to assess the overall treatment effect of the intervention. In addition, the models will be adjusted for age, ethnicity and level of education.

Regression coefficients (β), P values and 95% confidence intervals (CI) will be computed for all crude and adjusted mixed models. The statistical significance will be at $p<0.05$.

A Statistical Analyses Plan including details of statistical analyses will be finalised before dataset closure.

8.1 Primary study parameter(s)

Linear mixed model analyses will be performed to determine the effects of the preschool-based intervention on the primary outcome measure. Childcare organisation and preschool location will be added as random intercepts to take into account the clustered data structure. Overall models with treatment (intervention or control group) and the baseline value of a specific outcome measure will be made to assess the overall treatment effect of the intervention. In addition, the model will be adjusted for ECEC teachers' age, ethnicity and level of education.

8.2 Secondary study parameter(s)

Linear mixed model analyses will be performed to determine the effects of the preschool-based intervention on the secondary outcome measures. Childcare organisation and preschool location will be added as random intercepts to take into account the clustered

data structure. Overall models with treatment (intervention or control group) and the baseline value of a specific outcome measure will be made to assess the overall treatment effect of the intervention. In addition, the models will be adjusted for age, ethnicity and level of education.

8.3 Other study parameters

Descriptive statistics (n (%) or mean ± standard deviation) will be used to describe the characteristics of the study population.

9. ETHICAL CONSIDERATIONS

9.1 Regulation statement

The study will be conducted according to the principles of the Declaration of Helsinki (64th W MA General Assembly, Fortaleza, Brazil, October 2013) and in accordance with the Medical Research Involving Human Subjects Act (WMO) and other guidelines, regulations and Acts. The protocol needs yet to be approved by the Medical Ethics Committee (METC) of the VU University Medical Center.

9.2 Recruitment and consent

ECEC teachers will be informed about the research through their supervisor. In addition, the AUAS researchers will visit the locations to provide further information and hand out an information letter and the Informed Consent form. In times of corona, also digital meetings (via Microsoft Teams) will be used to inform ECEC teachers. With a minimum of 1 week time to consider their decision, AUAS researchers will visit (and in times of corona, call) the locations to answer any questions. If ECEC teachers then like to join the research, an Informed Consent in writing is obtained. In times of corona, it is still possible for the researchers to obtain written Informed Consent (the forms can be exchanged at the door). Parents will be informed of the AUAS researchers coming to the location and will be given a short heads up what the research is about by ECEC teachers. AUAS researchers then visit the locations to provide oral information about the research and will hand out written information and the Informed Consent form. Parents will be asked to read the information at home and consider joining the research with their child. The AUAS researchers will revisit the location 1 week after providing the information to answer any questions from the parents and if parents and their children like to join the research, an Informed Consent in writing is obtained. Parents give written permission for themselves and their child to participate. In times of corona, it is not possible to visit the preschools and talk to parents. Therefore, only via ECEC teachers some parents will be recruited. In times of corona, it is still possible for the researchers to obtain written Informed Consent (the forms can be exchanged at the door).

9.3 Objection by minors or incapacitated subjects

Code of conduct relating to expressions of objection by minors participating in medical research is applicable.
(https://english.ccmo.nl/investigators/publications/publications/2002/01/01/code-of-conduct-relating-to-expressions-of-objection-by-minors-participating-in-medical-research)

9.4 Benefits and risks assessment, group relatedness

Participants in the control group do not receive the intervention during the study. Benefits for ECEC teachers in the intervention group include the training (professional development) and Toolkit CO-HEALTHY which may improve the collaboration with parents regarding eating, physical (in)activity and sleeping behaviours of children. Both, the training and Toolkit are free of charge. Benefits for parents and children in the intervention group include the parent-child activities regarding eating, physical (in)activity and sleeping behaviours, which may improve the parent-child relationship and may have a positive effect on child development. The parent-child activities and related materials are free of charge.

The risks involving in this study are minimal. ECEC teachers are used to provide parent-child activities at preschools. In this study, specifically activities about eating, physical (in)activity and sleeping behaviours will be supervised. In total, 6 parent-child activities will be carried out by a local team of ECEC teachers during existing contact moments at preschools.

This study can only be performed by including children (minors, aged 2-4 years old), as the intervention is intended to reach children. It is aimed to examine the effects of the intervention on child level (eating behaviours/ physical (in)activity behaviours/sleeping behaviours/anthropometric measures). The risks and burden for children are minimal.

9.5 Compensation for injury

The sponsor/investigator has a liability insurance which is in accordance with article 7 of the WMO.

The sponsor (also) has an insurance which is in accordance with the legal requirements in the Netherlands (Article 7 WMO). This insurance provides cover for damage to research subjects through injury or death caused by the study.
The insurance applies to the damage that becomes apparent during the study or within 4 years after the end of the study.

9.6 Incentives

There is no financial compensation for participation in the study, neither for ECEC teacher nor for parents. The training and Toolkit /parent-child activities are free of charge.

10. ADMINISTRATIVE ASPECTS, MONITORING AND PUBLICATION

10.1 Handling and storage of data and documents

Data collection will be carried out by trained research staff and students of the AUAS. New quantitative and qualitative data will be collected at preschools with play-based education in Amsterdam. Data collected on paper questionnaires and score forms (with data of the height/weight/waist circumference measurements) will be entered twice by different staff/students (double data entry) in Microsoft Excel (.xlsx files). In times of corona, a digital version of the ECEC teacher questionnaire will be distributed via Qualtrics. The Qualtrics software is available for all employees of the AUAS and the Dutch AUAS-template in Qualtrics will be used. To protect the privacy of the participating ECEC teachers, an anonymous link to the Qualtrics questionnaire will be mailed to the private work e-mail address of ECEC teachers. In the first question of the digital version of the questionnaire, ECEC teachers will be asked to fill in their own participant number. Short interviews (with ECEC teachers and parents) and 24 hour dietary recalls will be recorded with Olympus Digital Voice Recorders (VN-731PC) and stored as .wma audio files. The short interviews will be held by telephone. The telephone will be set on speaker mode to make it possible to record the short conversations via the Digital Voice Recorders. After the interview, the noted telephone number of a parent will be destroyed. Pseudonymised transcripts will be made in Microsoft Word (.docx format). ActiLife software will be used to handle ActiGraph data (.agd/.csv files). IBM SPSS Statistics will be used for quantitative data analysis. MAXQDA will be used for qualitative data analysis.

All paper and electronic data files will be coded and therefore privacy of the participants will be protected:

- all locations will receive a unique location number (ascending, starting from 001);
- all ECEC teachers will receive a unique code starting with a location number followed by digit ‘1’ (to indicate it concerns an ECEC teacher) and a three-digit participant number (ascending, starting from 001);
- all parents will receive a unique code starting with a location number followed by digit ‘2’ (to indicate it concerns a parent) and a three-digit participant number (ascending, starting from 001). Children will receive the same code as their parents.

The key to the identification code will only be accessible for the executive AUAS researchers (S. Mul, N. Toussaint, M. Gündüz).

The handling of data will be dealt with in accordance to the EU General Data Protection Regulation and the Dutch Implementation Act of the General Data Protection Regulation (AVG/GDPR).

Coded paper data files (paper questionnaires and score forms) will be kept separate from signed Informed Consent forms. Both will be stored in locked filing cabinets in a room (B1.28) with swipe-card access at the Dokter Meurerhuis (AUAS).

Digital data files will be stored in folders on the Research Drive of the AUAS: a secure cloud storage service for research projects developed by SURF (SURFsara). A processor agreement between the AUAS and SURFsara exists. Files with personal data will be encrypted via VeraCrypt. Only specific AUAS researchers (S. Mul, M.T. Streppel, N. Toussaint, M. Gündüz) will have access to the folders. SURFdrive will be used for the storage of other digital files: a secure personal cloud storage service for Dutch education and research. Again, only specific AUAS researchers (S. Mul, M.T. Streppel, N. Toussaint, M. Gündüz) will have access to the folders.

After the study, paper documents are stored for 15 years in the archive of the Faculty of Sports and Nutrition (AUAS). Pseudonymised research data will be archived in the AUAS repository called Figshare.

10.2 Amendments

Amendments are changes made to the research after a favourable opinion by the accredited METC has been given. All amendments will be notified to the METC that gave a favourable opinion.

10.3 Annual progress report

The sponsor/investigator will submit the start date of the trial (date of inclusion of the first participant) to the accredited METC. The sponsor/investigator will submit a summary of the progress of the trial to the accredited METC once a year. Information will be provided on the date of inclusion of the first subject, numbers of subjects included and numbers of subjects that have completed the trial, serious adverse events/ serious adverse reactions, other problems, and amendments.

10.4 Temporary halt and (prematurely) end of study report

The investigator/sponsor will notify the accredited METC of the end of the study within a period of 8 weeks. The end of the study is defined as the last participants' last measurement.

The sponsor will notify the METC immediately of a temporary halt of the study, including the reason of such an action.

In case the study is ended prematurely, the sponsor will notify the accredited METC within 15 days, including the reasons for the premature termination.

Within 1 year after the end of the study, the investigator/sponsor will submit a final study report with the results of the study, including any publications/abstracts of the study, to the accredited METC.

10.5 Public disclosure and publication policy

The principal investigators will make decisions about the public disclosure and publication of the research data. All collected research data is property of the AUAS.

11. STRUCTURED RISK ANALYSIS

11.1 Potential issues of concern

Non other than described in section 9.4.

11.2 Synthesis

In accordance to section 10, subsection 1, of the WMO, the investigator will inform the participants and the reviewing accredited METC if anything occurs, on the basis of which it appears that the disadvantages of participation may be significantly greater than was foreseen in the research proposal. The study will be suspended pending further review by the accredited METC, except insofar as suspension would jeopardise the participants' health. The investigator will take care that all participants are kept informed. All test in this study are used in common practice, no special safety procedures are necessary. Tests are performed at preschools.

12. REFERENCES

1. NCD Risk Factor Collaboration (NCD-RisC). Worldwide trends in body-mass index, underweight, overweight, and obesity from 1975 to 2016: a pooled analysis of 2416 population-based measurement studies in 128.9 million children, adolescents, and adults. Lancet (London, England). 2017;390(10113):2627-42.
2. Labree LJ, van de Mheen H, Rutten FF, Foets M. Differences in overweight and obesity among children from migrant and native origin: a systematic review of the European literature. Obesity reviews : an official journal of the International Association for the Study of Obesity. 2011;12(5):e535-47.
3. de Hoog ML, van Eijsden M, Stronks K, Gemke RJ, Vrijkotte TG. Overweight at age two years in a multi-ethnic cohort (ABCD study): the role of prenatal factors, birth outcomes and postnatal factors. BMC public health. 2011;11:611.
4. Bouthoorn SH, Wijtzes AI, Jaddoe VW, Hofman A, Raat H, van Lenthe FJ. Development of socioeconomic inequalities in obesity among Dutch pre-school and school-aged children. Obesity (Silver Spring, Md). 2014;22(10):2230-7.
5. Atay Z, Bereket A. Current status on obesity in childhood and adolescence: Prevalence, etiology, co-morbidities and management. Obesity Medicine. 2016;3:1-9.
6. Singh AS, Mulder C, Twisk JW, van Mechelen W, Chinapaw MJ. Tracking of childhood overweight into adulthood: a systematic review of the literature. Obesity reviews : an official journal of the International Association for the Study of Obesity. 2008;9(5):474-88.
7. De Kroon ML, Renders CM, Van Wouwe JP, Van Buuren S, Hirasing RA. The Terneuzen birth cohort: BMI changes between 2 and 6 years correlate strongest with adult overweight. PloS one. 2010;5(2):e9155.
8. City of Amsterdam. Preschool and early childhood education. https://www.amsterdam.nl/en/education/preschool-education/.
9. Sisson SB, Krampe M, Anundson K, Castle S. Obesity prevention and obesogenic behavior interventions in child care: A systematic review. Preventive medicine. 2016;87:57-69.
10. Ward DS, Welker E, Choate A, Henderson KE, Lott M, Tovar A, et al. Strength of obesity prevention interventions in early care and education settings: A systematic review. Preventive medicine. 2017;95 Suppl:S37-S52.
11. van de Kolk I, Verjans-Janssen SRB, Gubbels JS, Kremers SPJ, Gerards S. Systematic review of interventions in the childcare setting with direct parental involvement: effectiveness on child weight status and energy balance-related behaviours. The international journal of behavioral nutrition and physical activity. 2019;16(1):110.
12. Dev DA, Byrd-Williams C, Ramsay S, McBride B, Srivastava D, Murriel A, et al. Engaging parents to promote children's nutrition and health: providers' barriers and strategies in head start and child care centers. American Journal of Health Promotion. 2017;31(2):153-62.
13. Toussaint N, Streppel MT, Mul S, Schreurs A, Balledux M, van Drongelen K, et al. A preschool-based intervention for Early Childhood Education and Care (ECEC) teachers in promoting healthy eating and physical activity in toddlers: study protocol of the cluster randomized controlled trial PreSchool@HealthyWeight. BMC public health. 2019;19(1):278.
14. Statistics Netherlands (CBS). CBS in uw buurt. http://www.cbsinuwbuurt.nl.
15. City of Amsterdam. Gezondheid in beeld. https://amsterdam.ggdgezondheidinbeeld.nl/.
16. Egert F, Dederer V, Fukkink RG. The impact of in-service professional development on the quality of teacher-child interactions in early education and care: A meta-analysis. Educational Research Review. 2020;29:100309.
17. Statistics Netherlands (CBS). Our services. Methodology. Classifications. Onderwijs en beroepen. Standaard Onderwijsindeling (SOI). Standaard Onderwijsindeling 2016. https://www.cbs.nl/nl-nl/onze-diensten/methoden/classificaties/onderwijs-en-beroepen/standaard-onderwijsindeling--soi--/standaard-onderwijsindeling-2016.
18. Statistics Netherlands (CBS). Our services. Methodology. Definitions. Migration background. https://www.cbs.nl/en-gb/our-services/methods/definitions?tab=m#id=migration-background.

19. Mech P, Hooley M, Skouteris H, Williams J. Parent-related mechanisms underlying the social gradient of childhood overweight and obesity: a systematic review. Child: care, health and development. 2016;42(5):603-24.
20. Stichting PAS. Monitor Samenwerken met ouders 2018-2019. https://www.stichtingpas.nl/publicaties/monitor-samenwerken-met-ouders-2018-2019/.
21. Dunst CJ, Trivette CM, Hamby DW. Technical Manual for Measuring and Evaluating Family Support Program Quality and Benefits. 2006.
22. IFIC. Food and Health Survey 2018. https://foodinsight.org/wp-content/uploads/2018/05/2018-FHS-Report-FINAL.pdf.
23. Gubbels JS, Sleddens EF, Raaijmakers L, Gies JM, Kremers SP. The Child-care Food and Activity Practices Questionnaire (CFAPQ): development and first validation steps. Public health nutrition. 2016;19(11):1964-75.
24. Sarphati Amsterdam. Sarphati Amsterdam collaboration policy 2019. https://sarphati.amsterdam/wp-content/uploads/2019/12/Sarphati-Amsterdam-collaboration-document_160919_DEF.pdf.
25. Musher-Eizenman D, Holub S. Comprehensive feeding practices questionnaire: validation of a new measure of parental feeding practices. Journal of pediatric psychology. 2007;32(8):960-72.
26. O’Connor TM, Cerin E, Hughes SO, Robles J, Thompson DI, Mendoza JA, et al. Psychometrics of the preschooler physical activity parenting practices instrument among a Latino sample. International journal of behavioral nutrition and physical activity. 2014;11(1):3.
27. Crawford DA, Ball K, Cleland VJ, Campbell KJ, Timperio AF, Abbott G, et al. Home and neighbourhood correlates of BMI among children living in socioeconomically disadvantaged neighbourhoods. British journal of nutrition. 2012;107(7):1028-36.
28. Fredriks AM, van Buuren S, Fekkes M, Verloove-Vanhorick SP, Wit JM. Are age references for waist circumference, hip circumference and waist-hip ratio in Dutch children useful in clinical practice? European journal of pediatrics. 2005;164(4):216-22.
29. ActiGraph. GT3X+ and wGT3X+ Device Manual 2013. https://s3.amazonaws.com/actigraphcorp.com/wp-content/uploads/2018/02/22094126/GT3X-wGT3X-Device-Manual-110315.pdf.
